# 35 years after CLIA 1988: Key insights and policy implications among laboratory professionals

**DOI:** 10.1371/journal.pone.0311251

**Published:** 2024-09-27

**Authors:** Jaime A. Nieto Sierra, David Gefen

**Affiliations:** Lebow College of Business, Drexel University, Philadelphia, Pennsylvania, United States of America; St John’s University, UNITED STATES OF AMERICA

## Abstract

The Clinical Laboratory Improvement Amendments (CLIA) regulations of 1988 required certification of some clinical laboratory professionals but not of others. Analyzing survey data 35 years later, we explore how laboratory professionals today are inadvertently affected by those regulations, specifically their sense of professional identity and their perceptions of justice—and the consequences of those on their turnover intentions. Turnover is a major concern among laboratory professionals. Survey results show that even 35 years after the unintended disenfranchisement caused by CLIA, clinical laboratory professionals whose specialty was included in CLIA have a stronger sense of being an ingroup, expressed as positive professional identity, and had a higher assessment of there being procedural and distributive justice than those excluded in CLIA. Turnover intentions, however, were primarily a matter of negative professional identity and reduced distributive justice.

## Introduction

In order to promote uniform quality and standards across laboratory testing sites, Congress enacted the Clinical Laboratory Improvement Amendments (CLIA) regulations of 1988 to outline specific laboratory performance indicators and to promote uniform quality and standards across all human laboratory testing sites in the United States. Refer to https://www.cdc.gov/clia/about.html. The regulations were supposed to apply to all laboratories dealing with health assessment or with diagnosing, preventing, or treating disease [1]. However, when CLIA was enacted and later revise by the Centers for Medicare & Medicaid Services (CMS) in 1992, it only included certain clinical laboratory professions and pathologists, leaving all other professions in the Anatomic Pathology (AP) laboratories out by omission. Among those included were specialties like clinical laboratory scientist (CLS)/medical laboratory technologist (MLT), pathologist, and cytotechnologist (CT). Among those omitted were histology technicians (HT), histotechnologists (HTL), pathologists’ assistants (PA) and Qualification in Immunohistochemistry (QIHC).

The CLIA categorization thus created a natural experiment setting that implied that certain laboratory professions are important enough to garner certification while others are not. Inadvertently, those regulations, by regulating some human specimen laboratory professions but not others, created an environment where professionals who perform regulated laboratory tests are treated differently from laboratory professionals who perform other tests, tests that were deemed at the time as pre-analytic processes and hence not regulated. Exacerbating that distinction, in many organizations, laboratory professionals who perform tests that were categorized as pre-analytic processes have less opportunity to be promoted.

Now, 35 years after CLIA, this study examines its long-term consequences on perceptions of justice, professional identity, and turnover intentions among laboratory professionals in view of social identity theory (SIT) [2, 3] and justice theory [4]. We chose those theories because prior research explicitly tied governmental policies to consequences of social identity [5], while other research tied justice to turnover [4].

### Social identity

According to social identity theory, people base their self-identity in part on the social group they believe that they are part of. People do so, argues the theory, even if that group is not real or belonging to it is irrational. The group a person believes he or she is part of is named the “ingroup” with all other people thought of as an “outgroup”. Once people feel that they are part of an ingroup they exacerbate any differences between their ingroup and the outgroup in an attempt to differentiate themselves positively, at least in their own minds, from all the others, i.e. what they perceive as the outgroup [6–8]. Social identity theory [2, 3] and its related social categorization theory [9] argue that social categorization into groups, even if done inadvertently or arbitrarily, also contributes to a sense of social identity and belongingness [2, 7, 9]. People, being self-motivated to think positively of themselves, then think more highly of people in their ingroup and disparagingly of those in the outgroup. That sense of being part of a “better” group then subjectively reflects positively on their own self-image. That overly positive assessment of the ingroup and negative assessment of the outgroup occurs even when there is no rational reason to justify it [2]. As a result, often, ingroup members look down at outgroup members, treating them with a negative bias while treating their perceived ingroup in an irrationally positive manner [7]. As research shows, it is enough to just superficially create groups to promote such as sense of social identity [10, 11]. Those social groups sometimes relate to existing social groups, such as soccer clubs or political parties, but they may be even arbitrarily set through randomly assigning people into groups [7].

Social identity also affects people’s attitudes [12], professional identity and pride [13], and ability to fulfill their roles [14]. Contrariwise, being made to feel outside of the ingroup, which may come about also by organizational policy or external regulations even when unintended, may negatively impact employees’ overall satisfaction, thus affecting the services they provide and increasing their dissatisfaction and resentment [13–15]. When people feel that they are an outgroup it may be manifested through their negative feelings of there being less justice, i.e., unfairness, and lower performance [16].

### CLIA and social identity

We suspect that CLIA inadvertently promoted such an ingroup-outgroup settings among laboratory professionals. That is, those required to be certified, having received a clear message from the authoritative governmental agency that their profession is important enough to garner certification would take that signal to heart and have a relatively stronger sense of a positive professional identity. That is, those regulated are expected to feel like an ingroup, having been set aside as having an implied more important (in that is it required to be certified) laboratory task than the others who are not regulated. In contrast, those not required to be certified, having received a clear message from the authoritative governmental agency that their profession is not important enough to garner certification would take that signal to heart and have a relatively stronger sense of a negative professional identity. Basically, laboratory professionals are expected to have responded rationally to the message made by the authoritative agency about the importance of their profession. Such an expected outcome is consistent with research that showed that governmental policies affect people’s sense of belonging within society [5, 17].

That consequence is consistent with SIT too. Specifically, the very act of treating one group of people as being special, in this case by holding them to a higher standard by requiring them to be certified, creates a sense of belonging to a special group, an ingroup. SIT tells us that once people are included into an ingroup they tend to feel that that group is special and better because believing so reflects positively on their self-identity [2, 7]. In this case, that self-identity is measured as positive professional identity. We do not posit that negative professional identity is an outcome of CLIA because it was never intended to treat down anybody, however, we do posit that negative professional identity will contribute to increased turnover intentions as noted by previous research too [18–20]. That is, and consistent with SIT, people prefer to have a positive self-image of themselves. Having a negative professional identity hardly contributes to achieving that, and so it is expected that also among laboratory professionals, having a negative professional identity will increase their intentions to leave their current place of employment.

### Social identity, justice, and turnover intentions

Additionally, being regulated, and hence having a certified procedure on how they conduct their laboratory tests, we expect the ingroup to feel that they have more say in how things are done, otherwise known as procedural justice [21]. According to Justice Theory, perceptions of justice / fairness play a central role in a wide variety of employee outcomes [21], including turnover intentions [22]. Key among those perceptions are procedural justice and distributive justice. Procedural justice is about “fairness of procedures used to determine the outcome distributions” [4, p. 425, 23–25]. Distributive justice is about “fairness of outcome distributions” [26–28]. According to Justice theory [21], low levels of procedural justice and distributive justice are key determinants of employee turnover [4]. Procedural and distributive justice are correlated [21], as are distributive justice and withdrawal behaviors [4, 16, 29]. Viewed in that manner, one of the consequences of CLIA should also be that those certified should have a higher sense of procedural justice expressed as feelings that they have more say on how things are done. That is also a rational response considering that they are held responsible by the authorities who certified them to uphold requirements that regulate how things are done.

As a footnote, it should be added that Justice Theory [21] discusses another two types of justice, namely interpersonal and informational justice. Interpersonal justice deals with being treated with dignity, politeness and respect. Informational justice deals with being provided with candid and open information and explanations in a timely manner. However, as there was no reason to assume that CLIA had anything to do with treating laboratory professionals differently in regard to interpersonal and informational justice based on their specialization, these two types of justice were dropped from the study in the interest of keeping the survey to within reasonable length. Likewise, there was no reason to assume so based on the experience of the lead author.

## Materials and methods

### Ethical considerations

The study was granted ethics approval by the Drexel University Office of Research & Innovation Institutional Review Board (protocol #2203009106) on April 13^th^, 2022. Upon clicking the survey invitation link, participants were presented with the consent form. Participants were informed that the study was approved by Drexel University IRB and were provided with the approval number and IRB contact information. Informed consent was obtained from all participants involved in this study via their clicking on a checkbox in the online survey, which indicated they have read the consent form, and they voluntarily agree to participate. Only subjects who checked that consent box were presented with the survey. Data were collected from consenting adults (18 and older) only.

### Research model

**Fig 1:**
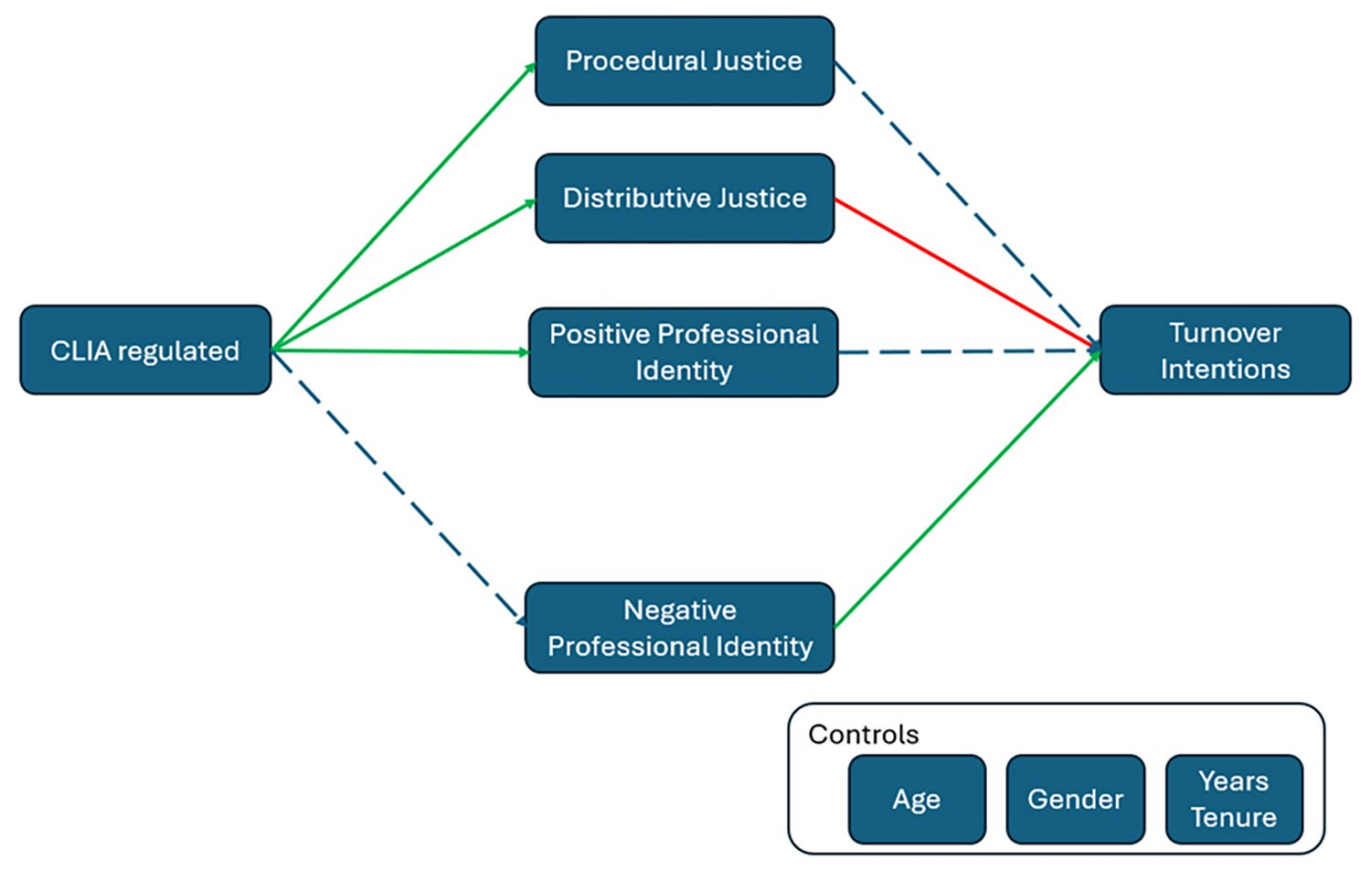
Research model. Positive effects are shown in green, negative in red, and insignificant in dashed lines. Correlations among ovals in the center and from the Controls to the other ovals are not shown.

CLIA created a pseudo-experimental design in which CLIA created a categorization of ingroup and non-ingroup laboratory professionals. A pseudo-experimental design is one where external circumstances create different treatment groups in real-world settings [30, 31]. The research model that details some of the subsequent consequences of that categorization in view of SIT and Justice Theory is shown in (Fig 1). The color coding and broken versus regular lines show the results of the data analysis. It is assumed that CLIA created an environment where certified laboratory professions have a positive professional identity. It is also assumed that that certification would affect perceptions of justice, and that those will affect turnover intentions. Adding SIT [10, 11], it was also assumed that the belief in being an ingroup member, expressed as positive professional identity, will also reduce turnover intentions because being part of the ingroup positively affects self-identity [2, 7]. Negative professional identity is assumed to increase turnover intentions.

### Study design

The data were purchased from Centiment in August 2022. Centiment surveyed relevant laboratory professionals in the United States. The dataset included 424 datapoints without missing values, 239 from lab professionals who are required to have CLIA certification and 185 who are not. All participants were adult laboratory professionals. Centiment is one of several panel data collection companies. Almost 20% of articles in leading management science journals used such services in 2020 [32]. Subjects were informed that their participation was voluntary and that they may stop their participation at any stage without penalty. Subjects checked a box on the online survey to indicate their consent.

### Data collection instrument

Procedural and distributive justice scales were adapted from the seminal paper by Colquitt [21]. Turnover intentions scales were adapted from Michaels and Spector [33]. Self-perception as an ingroup or not, measured as positive or negative professional identity, was adapted from the Macleod Clark Professional Identity Scale (MCPIS-9) in [34]. This scale has been extensively validated in research on medical professionals (e.g., [35–37]. The scale reflects either a sense of pride in being part of one’s profession or a sense of discontent with it. The items in that scale constitute our positive profession identity and negative profession identity items. On an exploratory basis, we also collected data on Self-assessed laboratory performance, adapted from Al-Dmour and Awamleh [38], but dropped that construct because in a preliminary principal components analysis (PCA) its items loaded together with positive profession identity. All the items used a 7-point Likert scale ranging from 1 “strongly disagree” to 7 “strongly agree.” Complete participant demographics are shown in Table 1. Some participants chose to skip those questions. Regarding gender, answers of “Non-binary / third gender” and “Prefer not to say” were replaced with missing values because there were too few such datapoints to allow analysis. All respondents were supposed to be above 18 years of age. We have no idea why two respondents answered that they are below 18 years of age. One respondent reported 478 years of experience–we changed that to a missing value as it is clearly a typo.

**Table 1:** Participant demographics.

		Frequency	Percent
Gender	Male	148	34.9
	Female	256	60.4
	Non-binary / third gender	9	2.1
	Prefer not to say	11	2.6
	Total	224	100.0
Age	Under 18	2	.5
	18–24	86	20.1
	25–29	114	26.9
	30–34	67	15.8
	35–44	83	19.6
	45–54	34	8.0
	55–64	20	4.7
	65–74	6	1.4
	75 or older	3	.7
	Prefer not to disclose	9	2.1
	Total	424	100.0
Race and ethnicity	White or of European descent	192	45.3
	Black or African American	119	28.1
	American Indian or Alaska Native	8	1.9
	Asian	32	7.5
	Native Hawaiian or Pacific Islander	8	1.9
	Mexican or Central American (Latin X) descent	39	9.2
	Other	11	2.6
	Prefer not to disclose.	15	3.5
	Total	424	100.0
Highest level of education you have completed	Less than high school	5	1.2
	High school graduate	30	7.1
	Some college	25	5.9
	2-year degree	44	10.4
	4-year degree	117	27.6
	Professional degree	134	31.6
	Doctorate	69	16.3
	Total	424	100.0
Your role/job level in your team	Team member	180	42.8
	Lead/Supervisor	107	25.2
	Manager	89	21.0
	Director	45	10.6
	Other. Please specify	3	0.7
	Total	424	100.0
Type of institution you currently work for	Non-profit hospital or healthcare system	65	15.3
	For-profit hospital or healthcare system	104	24.5
	Private, independent laboratory	72	17.0
	Reference laboratory	27	6.4
	VA hospital	37	8.7
	Research laboratory	75	17.7
	Veterinary laboratory	31	7.3
	Other. Please specify	13	3.1
	Total	424	100.0

## Data analysis

### Step 1. Confirmatory factor analysis

The data were analyzed using Mplus version 7.4 [39], a covariance-based structural equation modeling (CBSEM) package that enables analysis models in which there are many layers of dependent (predicted) and independent (predictor) variables and where the variables can also be latent and therefore measured through a collection of questionnaire items that reflect them (such as turnover intentions). As the data in Likert scale items are ordinal, an MLR estimation was run. MLR analyses are “robust to non-normality and non-independence of observations” [39, p. 533]. Simulations show that MLR may better control for type I errors (false positive) [40].

The analysis began by establishing the factorial validity of the model with a confirmatory factor analysis (CFA) [41]. The CFA model, which excluded the controls, has $\chi_{266}^{2}=433.199$, CFI = .96, TLI = .96, RMSEA = .039. Those are considered to show good overall model fit [42]. Sample size guidelines suggest that the sample size is adequate for CBSEM analysis [42], a recommendation that holds also based on recent simulation research on equivalent sized models to our one that showed little change in parameters estimated when increasing the sample size from 200 up to 500 [43]. (Mathematically, a perfect model should have an insignificant *χ*^2^ statistics, but even in the early days of CBSEM it was noted that assessments of model fit should not rely on that test alone because it is unrealistic [44, 45] and hence the recommendation is to look at all the fit indices when assessing model fit [42, 46]). Item loadings are practically the same as in the CBSEM model discussed next and so they are reported only there in Table 4. Having established the factorial validity of the model, we created averages of the items reflecting each of those constructs in SPSS and calculated their descriptive statistics. Those are shown in Table 2 by CLIA Certified professionals and the CLIA Non-Certified professionals and their t test comparisons. The correlations among those constructs and the controls as well as their descriptive statistics per the whole data are shown in Table 3. CLIA certification is 0 for lab professionals who are not required to have CLIA certification and 1 for those who are.

**Table 2:** Means and standard deviation by CLIA certified and non-certified respondents.

	CLIA certified		CLIA non-certified		T test (p-value)
	Mean	Std.	Mean	Std.	
Procedural Justice (PJ)	4.890	1.394	4.436	1.310	-3.404 (< .001)
Distributive justice (DJ)	4.844	1.547	4.330	1.530	-3.404 (< .001)
Ingroup (IN) as positive professional identity	5.301	1.197	4.889	1.358	-3.309 (< .001)
Negative professional identity (NPI)	3.808	1.846	3.919	1.705	.633 (.263)
Turnover intentions (TI)	3.460	1.609	3.771	1.524	2.013 (.022)

**Table 3:** Means and standard deviation and correlations for all the data.

	Mean	Std.	Correlations							
			PJ	DJ	IN	NPI	TI	CLIA	Age	Gender
PJ	4.693	1.375								
DJ	4.614	1.562	.626**							
IN	5.118	1.285	.688**	.644**						
NPI	3.859	1.785	.071	.144**	.016					
TI	3.597	1.577	-.060	-.133**	-.139**	.455**				
CLIA Certified	0.565	0.496	.164**	.164**	.159**	-.031	-.098*			
Age group	4.028	1.799	.087	.032	.123*	-.173**	-.085	.054		
Gender	1.726	0.642	.001	-.012	.047	-.012	.004	-.029	.046	
Years tenure	9.360	9.918	.043	-.006	.068	-152**	-.122*	.077	.583**	-.060

Significant * at the .05 level and ** at .01.

^a^ Age average is set as “Under 18” is 1, “18–24” is 2, etc.

### Step 2. CBSEM analysis

The research model was then run as a CBSEM model. This model includes the controls and specifies the paths among the constructs. This model has $\chi_{346}^{2}=541.881$, CFI = .96, TLI = .95, RMSEA = .037. Those are considered very good overall fit [42]. The model includes paths from CLIA classification to Procedural Justice, Distributive Justice, Positive Professional Identity, and Negative Professional Identity. The model also includes paths from those four constructs to Turnover Intentions, but not from CLIA. The Model also includes correlations among Procedural Justice, Distributive Justice, Positive Professional Identity, and Negative Professional Identity as well as correlations from the three controls to all the constructs. Table 4 shows the questionnaire items and their loadings. MLR does not produce standardized estimates, and so the first loading in each scale is set to 1 with a standard error (SE) set at 0. All the other item loadings are significant at the .001 level. As mentioned above, the Self-assessed laboratory performance items were dropped because in a preliminary PCA they loaded together with positive professional identity. The loading of the last item in the Turnover Intention scale is a bit lower than the others, but as dropping it produced essentially the same pattern as retaining it, and as it was part of the original scale, we retained it.

**Table 4:** Questionnaire items and their loadings.

Construct/Items Wording	Loading (SE)
**Procedural justice**	
I have been able to express my views and feelings during the creation or revision of our operating procedures	1.000(.000)
My thoughts and opinions were incorporated during the creation or revisions of the operating procedures	1.004(.048)
The operating procedures are applied consistently by everyone in the department	.971(.056)
The creation or revision of those procedures were free of bias	.846(.059)
The procedures are based on accurate information	.933(.063)
I have been able to speak up and appeal when the outcomes arrived by those procedures do not match the predicted outcomes	.930(.059)
I feel that the procedures uphold appropriate ethical and moral standards	.849(.068)
**Distributive justice**	
My pay reflects the effort I have put into my work	1.000(.000)
My pay is appropriate for the work I have completed	1.043(.048)
My pay reflects what I have contributed to the organization	1.017(.053)
My pay is justified given my performance	.942(.055)
**Positive Professional Identity**	
I feel like I am a member of my profession	1.000(.000)
I feel I have strong ties with members of my profession	.876(.049)
I am pleased to belong to my profession	.895(.053)
I can identify positively with members of my profession	.777(.062)
Being a member of my profession is important to me	.798(.064)
I feel I share characteristics with other members of my profession	.730(.063)
**Negative Professional Identity**	
I am often ashamed to admit that I am studying for my profession	1.000(.000)
I find myself making excuses for belonging to my profession	1.28(.056)
I try to hide that I am studying to be part of my profession	1.076(.054)
**Turnover Intentions**	
I intend to quit my current job	1.000(.000)
I have started to look for other jobs	1.173(.055)
I have often considered leaving my current job	1.111(.060)
I feel stuck in my current job and would move to a new career if I could	1.064(.066)
If I were to quit my job, I could find another career that is just as good	.610(.069)

The CBSEM model shows that Turnover Intentions were decreased by Distributive Justice (β = -.309, SE = .119, p-value = .009) and increased by Negative Professional Identity (β = .502, SE = .064, p-value < .001), but insignificantly affected by Procedural Justice (β = .150, SE = .107, p-value = .159) or by Positive Professional Identity (β = -.085, SE = .147, p-value = .563). Years Tenure, Age, Gender were insignificant (Γ = -.013, .074, .107, SE = .009, .051, .137, p-value = .141, .150, .438, respectively). We did not model a path from CLIA classification to Turnover Intentions because there was no theoretical reason to assume it. The saturated model analysis, discussed next, will show that that path is indeed insignificant.

In the rest of the model, Procedural Justice was increased by required CLIA classification (Γ = .448, SE = .145, p-value = .002) and was positively correlated with Distributive Justice (ψ = 1.298, SE = .153, p-value < .001), Positive Professional Identity (ψ = 1.481, SE = .144, p-value < .001), but not with Negative Professional Identity (ψ = .214, SE = .140, p-value = .126). Years Tenure, Age, Gender were insignificant (Γ = -.002, .088, .126, SE = .009, .054, .143, p-value = .829, .103, .381, respectively). Distributive Justice was increased by required CLIA classification (Γ = .530, SE = .159, p-value = .001) and was positively correlated with Positive Professional Identity (ψ = 1.473, SE = .156, p-value < .001) and Negative Professional Identity (ψ = .453, SE = .150, p-value = .002). Years Tenure, Age, Gender were insignificant (Γ = -.004, .014, .074, SE = .010, .057, .1350, p-value = .715, .801, .621, respectively). Positive Professional Identity was increased by required CLIA classification (Γ = .466, SE = .152, p-value = .002). It was not significantly correlated to Negative Professional Identity (ψ = .111, SE = .147, p-value = .451). Years Tenure, Age, Gender were insignificant (Γ = .001, .093, .229, SE = .010, .061, .155, p-value = .915, .125, .140, respectively). Negative Professional Identity was insignificantly affected by required CLIA classification (Γ = -.070, SE = .165, p-value = .670). Years Tenure, Age, Gender were insignificant (Γ = -.020, -.072, -.028, SE = .011, .066, .159, p-value = .058, .274, .862, respectively).

Replicating this model as a maximum likelihood model to produce R^2^ values ($\chi_{346}^{2}=688.771$, CFI = .94, TLI = .93, RMSEA = .050) provides an equivalent pattern of significant paths with R^2^ values being Procedural Justice 4%, Distributive justice 3%, Positive professional identity 5%, Negative professional identity 4%, and Turnover intentions 31%. Replicating the model by down-sampling so there are an equivalent number of datapoints from those required as from those not requited to have CLIA certification also produced equivalent results.

### Nested models

To test nested models, and in accordance with [41], we added a saturated model to the analysis, showing that the paths we did not include are indeed insignificant and that the Δ*χ*^2^ is likewise insignificant. A saturated model adds all possible paths in the model including those that were not originally thought to be relevant. The saturated model has $\chi_{345}^{2}=562.457$, meaning that the $\Delta \chi_{1}^{2}=1.317$ is insignificant (p-value = .251) and the added path from CLIA classification of Turnover Intentions is insignificant (Γ = -.158, SE = .144, p = .270). Checking further by running a minimal model that excludes all the insignificant paths produces $\chi_{348}^{2}=565.283$, meaning that the $\Delta \chi_{2}^{2}=1.509$ is insignificant (p = .219).

### Other robustness checks

Testing whether institution type (Non-profit hospital or healthcare system, For-profit hospital or healthcare system, Private, independent laboratory, Reference laboratory, VA hospital, Research laboratory, Veterinary laboratory, Other) or organizational role (Team member, Lead/Supervisor, Manager, Director, Other) have any effect, we first ran a general linear model (GLM) with the constructs in the model as the dependent variables and institution type and organizational role as fixed factors. The constructs for the GLM analyses were created as the averages of the items that reflect each construct in the CFA, exactly as in Tables 2 and 3. Research suggests that analysis of variance, regression, and correlation methods are robust to violation of assumed normal distribution [47].

Institution type had an insignificant effect (Pillai’s Trace F statistic = 1.220, p-value = .035) with Procedural Justice insignificantly affected (F = 1.295, p-value = .251), Distributive Justice marginally significantly (F = 2.382, p-value = .021), Positive Professional Identity insignificantly (F = 1.731, p-value = .100), Negative Professional Identity insignificantly (F = .772, p-value = .611), Turnover Intentions insignificantly (F = 1.303, p-value = .247), and CLIA certification significantly (F = 2.860, p-value = .006). In contrast, organizational role was insignificant (Pillai’s Trace F statistic .573, p-value .951), with Procedural Justice, Distributive Justice, Positive Professional Identity, Negative Professional Identity, Turnover Intentions, and CLIA certification all insignificant (F = .178, 1.325, .870, .448, .604, .166, p-values = .950, .260, .482, .774, .660, .956, respectively).

Running a GLM to replicate the CBSEM model with Turnover Intentions as the dependent variable produced a significant model (F = 5.287, p-value < .001, R^2^ = .30), where the fixed factors Institution Type, Organizational Role, gender, education level, ethnicity are insignificant (F = .774, .283, .148, 1.532, 1.310, p-value = .609, .595, .931, .166, .244, respectively), as are the covariates Years Tenure (borderline), Procedural Justice, Positive Professional Identity, and age (F = 4.123, 2.426, 1.231, 2.591, p-value = .043, .120, .271, .108, respectively). It is predicted by Distributive Justice (F = 10.892, p-value = .001, β = -.210) and Negative Professional Identity (F = 197.186, p-value < .001, β = .406), producing a significance pattern reminiscent of the CBSEM.

## Discussion

CLIA regulations created a natural quasi-experiment that separated the clinical laboratory workforce into two groups. This study shows the unintended and unexpected consequences of that action on procedural and distributive justice and on positive and negative professional identification, and the effect of those on turnover intentions. The results are mostly consistent with social identity theory and justice theory, but also indirectly suggest steps that could be taken to reduce turnover. Separating the workforce into two groups and recognizing one but not the other 35 years ago is correlated with a current stronger sense of positive professional identity among those in the chosen clinical laboratory professions. That is reminiscent of an ingroup in SIT and may suggest that CLIA created an *esprit de corps*, as evident in the positive professional identity of those recognized to be certified. That classification was also highly correlated with increased procedural and distributive justice. Procedural and distributive justice besides being highly correlated with each other as suggested by Justice Theory are also both highly correlated with positive professional identity, suggesting that the natural quasi-experiment that separated the clinical laboratory workforce into two groups also indirectly reduced turnover intentions through both distributive justice and the correlation between distributive justice and procedural justice and positive professional identity.

The higher perceptions of procedural and distributive justice and positive professional identity among those professionals performing those laboratory tests selected in CLIA could be a rational response. Being told that what you do is important should arguably contribute to positive professional identity, and having your legal responsibilities clarified through the certification process should encourage people to be more involved in how their laboratory tests are run and as a consequence increase their procedural justice. Adding an SIT perspective suggests another reason. Being an ingroup member, people feel they have more at stake because according to SIT their subjective sense of importance is tied to their ingroup status. Having a psychological stake in the game as an ingroup member, it would be expected that ingroup members will also have a stronger need to be involved, and hence being more involved will have a higher feeling of procedural justice. Those ingroup members might also be expected to have a higher approval of their pay, i.e. distributive justice, considering it is “their” group rather than just doing a job for someone else. That CLIA had only an indirect effect on turnover intentions implied that its effects are mediated as SIT and Justice Theory imply. That may be why the correlations among distributive justice, procedural justice, and positive professional identity are so high. That Institution Type, Organizational Role, gender, education level, ethnicity, and Years Tenure made no significant difference is telling about how strong those relationships are. That not being selected by CLIA did not affect negative professional identity maybe because of the time elapsed since CLIA and hence that many of the respondents joined their profession long after CLIA, see Table 3, and so its impact may have been blunted over the years, even assuming they even knew of that history. Or it might just be that, contrary to our initial assumptions, while CLIA sent a message of how important you are to some laboratory professionals by their inclusion, it did not send a negative signal to the others by their exclusion. That may also be the reason that positive and negative professional identity are not significantly correlated.

These results highlight healthcare industry challenges and the role that governmental regulations play in influencing employees’ environments and behavior. Although industry structures are beyond management’s control, leaders may devise other internal structures to create an *esprit de corps* also among laboratory professionals who are not certified by CLIA. Laboratory managers may want to pay close attention to the negative professional identity some of their professionals might harbor because, even outside the realm of certification and its consequences, that is what is most highly correlated with turnover intentions. SIT might suggest a mechanism for doing so through making those professionals feel that they are part of a select team. As that ingroup perception is highly correlated with procedural justice, and hence presumably with better testing through more involvement of the laboratory professionals in the process, the consequences may be telling. As is, there are high healthcare costs and error rates in pathology diagnostic services and the economic and other burdens of that on healthcare systems, patients, insurance, and other payers can be substantial [48]. With, for example, reports of error rates in cancer diagnosis varying from 5% [49], to 11.8% [50], and up to 20% [51] and prefinal diagnostic errors as high as 58.4% [52], the impact of more involved laboratory professionals could be substantial.

### Limitations

A key limitation is the cross-sectional nature of the survey, which can establish a measure of association but not causation. Moreover, as is to be expected from survey research, there might always be additional variables of interest that could have been included, even if this study did center on justice, which some of the most cited papers on employee turnover center on (e.g., [21], as we did too.

## Conclusions

Viewing the consequences of CLIA 35 years later on, the inadvertent creation of regulated vs. unregulated anatomic pathology professions still has lasting impacts and may suggest steps that could be taken to reduce turnover intentions. Even after 35 years, being included in the select group of professionals who, because of the laboratory tests they perform, are required to be certified still creates a higher sense of positive professional identity as well as increased perceived procedural and distributive justice. That increased distributive justice is correlated with reduced turnover intentions. Still, the most impactful correlation with turnover intentions is negative professional identity. Interpreting the findings through the theoretical lenses of SIT might suggest ways to reduce negative professional identity.
